# Supplementary material for: Distinguishing the Effects of Water Volumes versus Stocking Densities on the Skeletal Quality during the Pre-Ongrowing Phase of Gilthead Seabream (Sparus aurata)
Source: Animals (Basel). 2023 Feb 5;13(4):557. doi: 10.3390/ani13040557 (PMC9951685; doi:10.3390/ani13040557)
Supplement: Supplementary file 1 [file animals-13-00557-s001.zip › animals-2185709-supplementary.pdf]

Supplementary Materials

Zachary Dellacqua [1,2,*], Claudia Di Biagio [1,3], Corrado Costa [4], Pedro Pousão-Ferreira [5], Laura Ribeiro [5], Marisa Barata [5], Paulo J. Gavaia [6], Francesco Mattei [7], Andrea Fabris [8], Marisol Izquierdo [2] and Clara Boglione [1]

1Department of Biology, University of Rome 'Tor Vergata', 00133 Rome, Italy
2 Ecoaqua Institute, University of Las Palmas de Gran Canaria, 35214 Telde, Gran Canaria, Spain
3 Laboratory of Evolutionary Developmental Biology, University of Ghent, 9000 Ghent, Belgium
4 CREA—Consiglio per la Ricerca in Agricoltura e L'analisi Dell'economia Agraria (CREA)—Centro di Ricerca Ingegneria e Trasformazioni Agroalimentari, 00015 Rome, Italy
5 IPMA—Instituto Portugues do Mar e Atmosfera—Research Station, 8700-305 Olhão, Portugal
6 CCMAR—Centre of Marine Sciences, University of the Algarve, 8005-139 Faro, Portugal
7 UMR 7093, Laboratoire d'Oceanographie de Villefranche (LOV), Sorbonne University, 06230 Villefranche-sur-Mer, France
8 Associazione Piscicoltori Italiani, 37135 Verona, Italy
***** Correspondence: dellacquaz95@gmail.com; Tel.: +39-351-857-0196

Supplementary Table S1. Pre-ongrowing rearing established tank densities and performative parameters

	**Initial setup (August 2nd)**			**1st sampling (August 29th)**				**2nd sampling (September 18th)**				**Final sampling (October 4th)**		
**Conditions**	**Initial number of fish**	**Initial weight/ fish (g)**	**Initial density (kg/m^3)**	**Weight/ fish (g)**	**New tank density (kg/m^3)**	**Number of fish after removal**	**Density established after removal of fish (kg/m^3)**	**Weight/ fish (g)**	**Current tank density**	**Number of individuals after removal**	**Density established after removal of fish**	**Measured seabream individuals**	**Final average weight**	**Final average TL (cm)**
**LD 1000L**	370	9.0	3.3	19.6	7.2	255	5	47.3	12.0	148	7	66	56.0 ± 14.8	14.8 ±1.2
**MD 1000L**	760	8.7	6.6	19.1	14.5	525	10	41.4	21.7	338	14	89	57.6 ± 16.2	14.5 ± 1.5
**HD 1000L**	1460	9.0	13.2	19.7	27.1	1017	20	40.6	41.1	690	28	179	54.3 ± 15.2	14.1 ± 1.3
**LD 500L Tank 1***	183	8.7	3.2	20.1	7.3	125	5	43.8	10.9	80	7	71	54.8 ± 13.9	15.1± 1.2
**LD 500L Tank 2***	187	8.6	3.2	21.1	7.9	118	5	42.0	9.9	83	7	74	53.3 ± 11.5	14.7±1.1
**MD 500L**	380	8.6	6.6	20.6	15.7	343	10	42.8	20.8	164	14	115	55.4 ± 12.8	14.9 ± 1.1
**HD 500L**	740	8.9	13.2	20.1	29.5	497	20	39.6	39.3	353	28	214	52.0 ± 15.3	14.4 ±1.3

*Two tanks of LD500 were used in order to acquire sufficient final number of individuals for statistical comparisons

a)

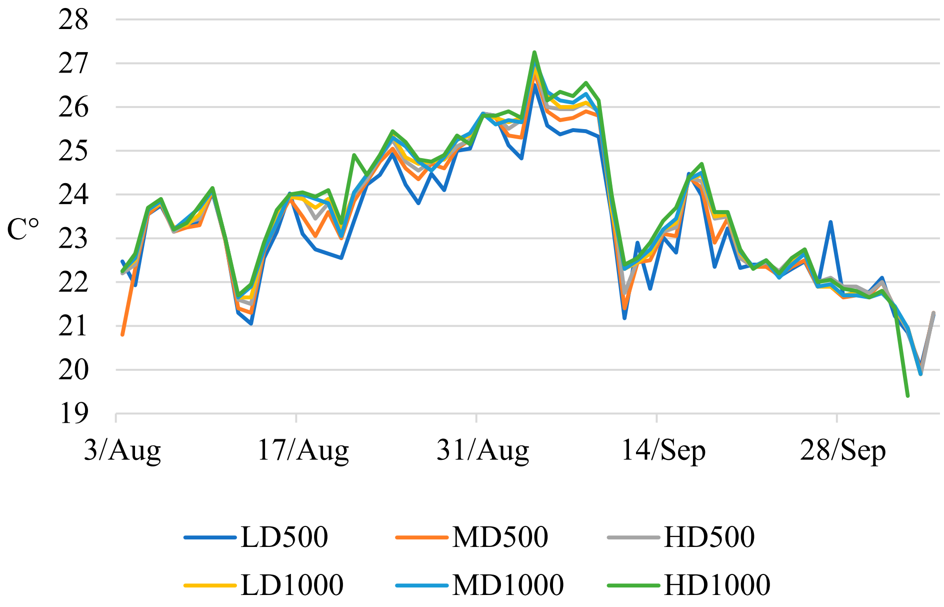

b)

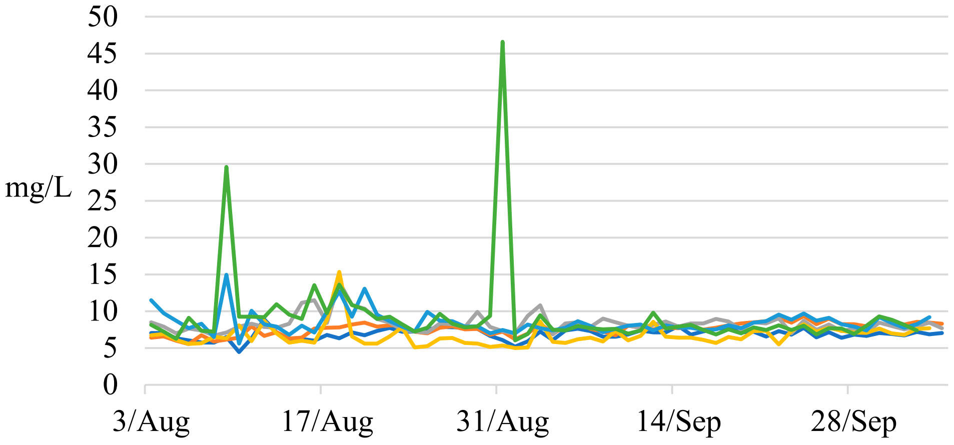

Supplementary Figure S1. a) *Average daily temperature throughout rearing.* ***b)*** *Average daily dissolved oxygen throughout rearing.*

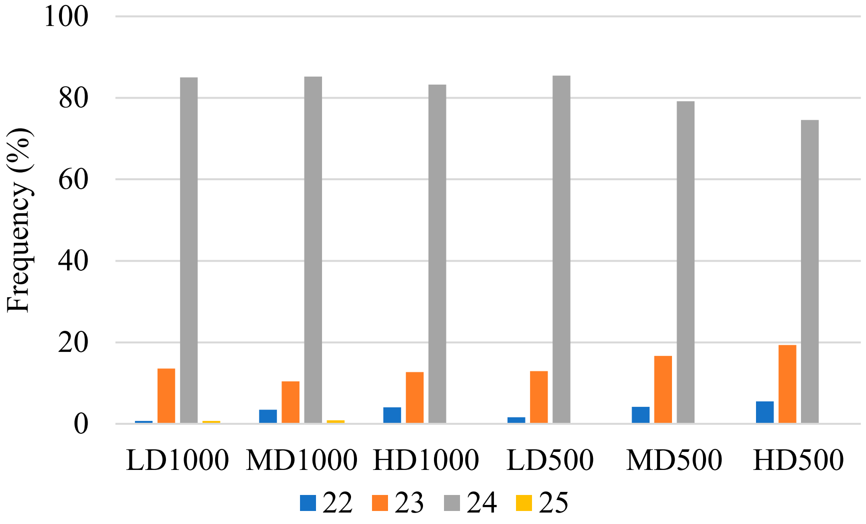

Supplementary Figure S2. *Frequency classes of vertebrae counts in the experimental lots.*

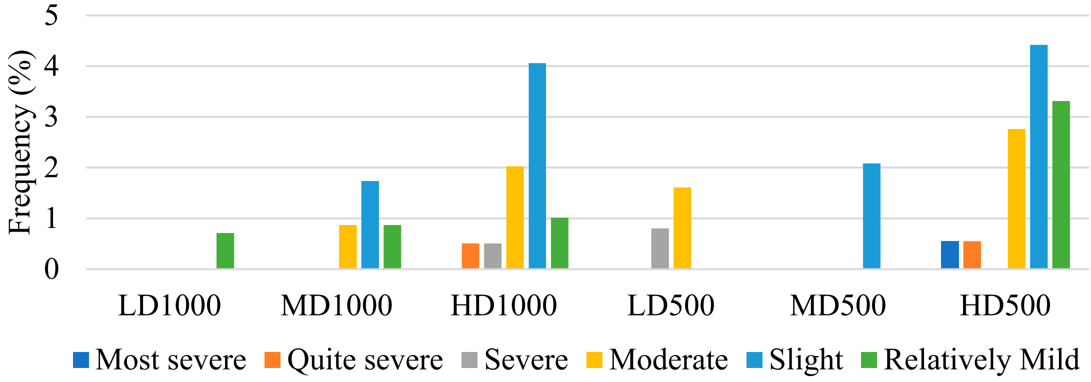

Supplementary Figure S3. *Distribution of different angular classes of kyphotic curvatures among the density and volume conditions.*

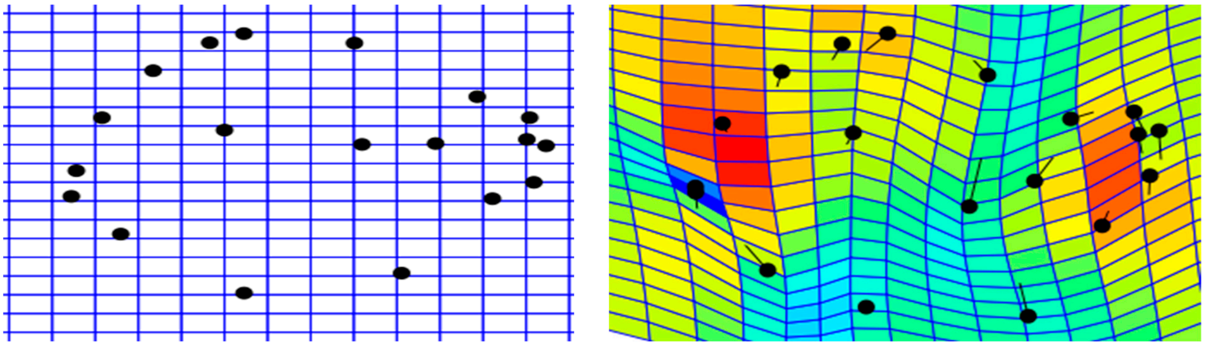

Supplementary Figure S4. *Normal LD1000 gilthead seabream TPS on left and lordotic HD500 gilthead seabream TPS on right.*

Supplementary Table S2. List of considered anomaly types

Region	
**A**	Cephalic vertebrae
**B**	Abdominal vertebrae
**C**	Hemal vertebrae
**D**	Caudal vertebrae
Type	
**1**	Kyphosis
**2**	Lordosis
**3**	Vertebral centra partial or complete fusion
**4**	Vertebral shape anomaly
**5**	Underbite
**6**	Overbite
**7**	Opercular plate anomaly

Supplementary Table S3. Relative frequency of specific skeletal anomalies among the conditions

%	LD1000	MD1000	HD1000	LD500	MD500	HD500	T_0
A1	0.00	0.00	0.00	0.00	0.00	0.00	0.00
A2	0.00	0.87	0.51	0.00	1.04	2.21	0.62
A3	0.00	0.00	0.00	0.00	1.04	0.00	1.24
A4	0.71	0.00	1.02	2.42	2.08	0.00	10.56
A/B1	0.00	0.00	1.02	0.00	0.00	1.10	1.24
A/B2	1.43	0.00	0.00	0.00	0.00	0.55	0.62
A/B3	0.00	0.87	0.00	0.00	0.00	0.00	1.24
B1	0.00	1.74	4.57	2.42	1.04	3.31	0.62
B2	0.71	3.48	2.54	0.81	4.17	3.31	6.21
B3	4.29	5.22	2.54	1.61	4.17	2.21	11.80
B4	7.86	3.48	3.05	4.84	3.13	1.10	37.27
B/C1	0.00	0.00	0.00	0.00	0.00	0.00	0.62
B/C2	2.86	3.48	6.09	2.42	4.17	12.71	1.86
B/C3	0.71	0.00	2.54	0.81	2.08	1.66	0.00
C1	0.00	0.00	1.02	0.00	0.00	3.31	0.00
C2	26.43	29.57	40.61	25.00	27.08	29.83	29.81
C3	1.43	5.22	3.55	5.65	4.17	2.76	4.35
C4	14.29	11.30	10.15	18.55	12.50	11.60	83.85
C/D1	0.71	1.74	1.52	0.00	1.04	2.21	0.00
C/D2	2.14	0.00	3.55	3.23	1.04	2.21	0.62
C/D3	1.43	0.87	1.02	0.00	1.04	2.21	0.00
D1	0.00	0.00	1.02	1.61	0.00	1.10	0.00
D2	0.00	0.87	1.52	0.00	0.00	1.66	0.00
D3	1.43	0.87	4.06	1.61	3.13	2.21	1.24
D4	3.57	1.74	3.55	2.42	4.17	3.31	17.39
5	24.29	25.22	30.96	29.84	31.25	36.46	22.98
6	1.43	6.96	7.11	4.03	5.21	8.84	12.42
7	5.71	8.70	18.78	15.32	17.71	23.20	16.77

Supplementary Table S4. Frequency of individuals exhibiting specific skeletal anomalies among the conditions

%	LD1000	MD1000	HD1000	LD500	MD500	HD500	T_0
A1	0.00	0.00	0.00	0.00	0.00	0.00	0.00
A2	0.00	0.87	0.51	0.00	1.04	2.21	0.62
A3	0.00	0.00	0.00	0.00	1.04	0.00	1.24
A4	0.71	0.00	0.51	1.61	1.04	0.00	8.70
A/B1	0.00	0.00	1.02	0.00	0.00	1.10	1.24
A/B2	1.43	0.00	0.00	0.00	0.00	0.55	0.62
A/B3	0.00	0.87	0.00	0.00	0.00	0.00	1.24
B1	0.00	1.74	4.57	2.42	1.04	3.31	0.62
B2	0.71	3.48	2.54	0.81	4.17	3.31	6.21
B3	4.29	5.22	2.54	1.61	3.13	2.21	10.56
B4	3.57	2.61	1.52	2.42	1.04	0.55	19.25
B/C1	0.00	0.00	0.00	0.00	0.00	0.00	0.62
B/C2	2.86	3.48	6.09	2.42	4.17	12.71	1.86
B/C3	0.71	0.00	1.52	0.81	1.04	1.10	0.00
C1	0.00	0.00	1.02	0.00	0.00	3.31	0.00
C2	26.43	29.57	40.61	25.00	27.08	29.83	29.81
C3	1.43	3.48	3.55	4.84	3.13	2.76	4.35
C4	7.14	6.09	6.60	8.06	7.29	7.73	34.78
C/D1	0.71	1.74	1.52	0.00	1.04	2.21	0.00
C/D2	2.14	0.00	3.55	3.23	1.04	2.21	0.62
C/D3	1.43	0.87	1.02	0.00	1.04	2.21	0.00
D1	0.00	0.87	1.52	0.00	0.00	1.66	0.00
D2	0.00	0.00	1.02	1.61	0.00	1.10	0.00
D3	1.43	0.87	4.06	1.61	3.13	2.21	1.24
D4	3.57	1.74	3.05	2.42	3.13	2.76	13.66
5	24.29	25.22	30.96	29.84	31.25	36.46	22.98
6	1.43	6.96	7.11	4.03	5.21	8.84	12.42
7	5.71	8.70	18.78	15.32	17.71	23.20	16.77

Supplementary Table S5. Descriptors used to preform PCA ordination model (see Sup. Tab. 2 for the codes of the anomalies).

PCA					
Kyphosis	Lordosis	Fusion	Underbite	Overbite	Operculum
A1<br>B1<br>C1<br>D1	A2<br>B2<br>C2<br>D2	A3<br>B3<br>C3<br>D3	5	6	7
